# Supplementary material for: Rhizobia Isolated from the Relict Legume Vavilovia formosa Represent a Genetically Specific Group within Rhizobium leguminosarum biovar viciae
Source: Genes (Basel). 2019 Dec 1;10(12):991. doi: 10.3390/genes10120991 (PMC6947851; doi:10.3390/genes10120991)

Table S1. List of strains, their hosts, isolation sites and the accession numbers of genes used in the study. * data from this study.

Strain	Host	Origin	16S rRNA	*dna*K	*glt*A	*gln*II	*nod*A	*nod*C	*nod*D	*nif*H	*nod*X
Rl Vaf-01*	*Vavilovia formosa*	N. Ossetia (Russia)	KF662879	MK676096	MK676118	KY742626	KY930250	KF753618	KY930298	KY930225	MK676140
Rl Vaf-09*	*Vavilovia formosa*	N. Ossetia (Russia)	KF662881	MK676097	MK676119	KY742628	KY930252	KF753620	KY930300	KY930227	MK676141
Rl Vaf-10*	*Vavilovia formosa*	N. Ossetia (Russia)	KF662882	MK676098	MK676120	KY742629	KY930253	KF753621	KY930301	KY930228	MK676142
Rl Vaf-11*	*Vavilovia formosa*	N. Ossetia (Russia)	KF662883	MK676099	MK676121	KY742631	KY930255	KF753622	KY930304	KY930230	MK676143
Rl Vaf-12*	*Vavilovia formosa*	N. Ossetia (Russia)	KF662884	MK676100	MK676122	KY742632	KY930258	KF753623	KY930307	KY930233	MK676144
Rl Vaf-13*	*Vavilovia formosa*	N. Ossetia (Russia)	KF662885	MK676101	MK676123	KY742633	KY930262	KF753624	KY930311	KY930237	MK676145
Rl Vaf-14*	*Vavilovia formosa*	N. Ossetia (Russia)	KF662886	MK676102	MK676124	KY742634	KY930264	KF753625	KY930313	KY930239	MK676146
Rl Vaf-23*	*Vavilovia formosa*	N. Ossetia (Russia)	KF662887	MK676103	MK676125	KY742635	KY930265	KF753626	KY930314	KY930240	MK676147
Rl Vaf-25*	*Vavilovia formosa*	N. Ossetia (Russia)	KF662889	MK676104	MK676126	KY742636	KY930267	KF753628	KY930316	KY930242	MK676148
Rl Vaf-26*	*Vavilovia formosa*	N. Ossetia (Russia)	KF662890	MK676105	MK676127	KY742637	KY930268	KF753629	KY930317	KY930243	MK676149
Rl Vaf-45*	*Vavilovia formosa*	Armenia	KY742614	MK676106	MK676128	KY742638	KY930269	KY930274	KY930318	KY930244	MK676150
Rl Vaf-46*	*Vavilovia formosa*	Armenia	KY742616	MK676107	MK676129	KY742639	KY930270	KY930275	KY930319	KY930245	MK676151
Rl Vaf-51*	*Vavilovia formosa*	Armenia	KY742617	MK676108	MK676130	KY742640	KY930271	KY930276	KY930320	KY930246	MK676152
Rl Vaf-72*	*Vavilovia formosa*	Armenia	KY742615	MK676109	MK676131	KY742642	KY930273	KY930278	KY930322	KY930248	MK676153
Rl Vaf-96*	*Vavilovia formosa*	Dagestan (Russia)	KY742618	MK676110	MK676132	KY742643	KY930266	KY930290	KY930303	KY930249	MK676154
Rl Vaf-108*	*Vavilovia formosa*	Dagestan (Russia)	KY742619	MK676111	MK676133	KY742630	KY930254	KY930291	KY930302	KY930229	MK676155
Rl Vaf-114*	*Vavilovia formosa*	Dagestan (Russia)	KY742620	MK676112	MK676134	KY742644	KY930256	KY930292	KY930305	KY930231	MK676156
Rl Vaf-119*	*Vavilovia formosa*	Dagestan (Russia)	KY742621	MK676113	MK676135	MH449645	KY930257	KY930293	KY930306	KY930232	MK676157
Rl Vaf-125*	*Vavilovia formosa*	Dagestan (Russia)	KY742622	MK676114	MK676136	KY742645	KY930259	KY930294	KY930308	KY930234	MK676158
Rl Vaf-127*	*Vavilovia formosa*	Dagestan (Russia)	KY742623	MK676115	MK676137	KY742646	KY930260	KY930295	KY930309	KY930235	MK676159
Rl Vaf-129*	*Vavilovia formosa*	Dagestan (Russia)	KY742624	MK676116	MK676138	KY742647	KY930261	KY930296	KY930310	KY930236	MK676160
Rl Vaf-137*	*Vavilovia formosa*	Dagestan (Russia)	KY742625	MK676117	MK676139	KY742648	KY930263	KY930297	KY930312	KY930238	MK676161
Rl bv. *viciae* 248	*Vicia faba*	UK	ARRT01000001-ARRT01000007								-
Rl bv. *viciae* 3841	*Pisum sativum*	UK	NC_008380				NC_008381				-
Rl bv. viciae BIHB 1217	*pea*	India	NZ_CP022665				NZ_CP022669				-
Rl bv. viciae GB30	*Pisum sativum*	Poland	ATTP01000001-ATTP01000078								-
Rl bv. *viciae* TOM	*Pisum sativum Afg*	Turkey	AQUC01000001-AQUC01000006								
Rl bv. *viciae* UPM1131	*Pisum sativum*	Italy	ATYZ01000001-ATYZ01000042								-
Rl bv. *viciae* UPM1137	*Pisum sativum*	Italy	ATYN01000001-ATYN01000053								-
Rl bv. viciae UPM791	*Pisum sativum*	USA	NZ_CP025509				NZ_CP025505				-
Rl bv. *viciae* USDA 2370	*Pisum sativum*	USA	MRDL01000001-MRDL01000072								-

Rl bv. viciae Vc2	*Vicia cracca*	UK	ARDP01000001-ARDP01000143		
Rl bv. viciae VF39	*Vicia faba*	Germany	ATYQ01000001-ATYQ01000090		-
Rl bv. *viciae* WSM1455	*Vicia faba*	Greece	AJUF01000001-AJUF01000035		-
Rl bv. *viciae* WSM1481	*Lens culinaris*	Greece	AQUM01000001-AQUM01000006		-
Rl ATCC 14479	*Trifolium praetense*	USA	NZ_CP030760	NZ_CP030762	
Rl bv. *trifolii* CB782	*Trifolium semipilosum*	Kenya	NZ_CP007067	NZ_CP007070	
Rl bv. trifolii CC275e	*Trifolium repens*	Australia	JRXL01000001-JRXL01000028		
Rl bv. trifolii CC278f	*Trifolium nanum*	USA	AJUI01000001-AJUI01000052		
Rl bv. trifolii CC283b	*Trifolium ambiguum*	Caucasus (Russia)	AZXS01000001-AZXS01000109		
Rl bv. trifolii Rt24.2	*Trifolium pratense*	Poland	MAMO01000001-MAMO01000179		
Rl bv. trifolii SRDI565	*Trifolium subterraneum*	Australia	AQUD01000001-AQUD01000007		
Rl bv. *trifolii* SRDI943	*Trifolium subterranium*	Australia	AQUN01000001-AQUN01000005		
Rl bv. trifolii TA1	*Trifolium spumosum*	Australia	AKIA01000001-AKIA01000037		
Rl bv. *trifolii* WSM1325	*medditerian clovers*	Greece	NC_012850	NC_012848	
Rl bv. *trifolii* WSM1689	*Trifolium uniflorum*	Greece	NZ_CP007045	NZ_CP007048	
Rl bv. *trifolii* WSM2012	*Trifolium rueppellianum*	Ethiopia	AJUJ01000001-AJUJ01000068		-
Rl bv. *trifolii* WSM2297	*Trifolium africanum*	S. Africa	AJUK01000001-AJUK01000030		-
Rl bv. *trifolii* WSM2304	*Trifolium polymorphum*	S. America	NC_011369	NC_011368	
Rl bv. *trifolii* WSM597	*Trifolium pallidum*	S. America	AKHZ01000001-AKHZ01000053		

Table S2. Group separation (in %) for groups *Vaf*, *Rlv* and *Rlt*, calculated in Bionumerics using Jackknife (maximum similarities). Intensity of colour corresponds to the value.

Gene		Group separation (JK)		
		Vaf	*Rlv*	*Rlt*
16s rRNA	*Vaf*	55,3	19,23	38,89
	Rlv	14,39	61,54	32,22
	Rlt	30,3	19,23	28,89
*dna*K	*Vaf*	86,36	17,95	8,89
	Rlv	13,64	52,56	32,22
	Rlt	0	29,49	58,89
*gln*II	*Vaf*	88,64	3,85	20
	Rlv	0	69,23	30
	Rlt	11,36	26,92	50
*glt*A	*Vaf*	79,55	3,85	15,56
	Rlv	9,09	73,08	38,89
	Rlt	11,36	23,08	45,56
*nod*A	*Vaf*	100	15,38	0
	Rlv	0	84,62	0
	Rlt	0	0	100
*nod*C	*Vaf*	100	23,08	0
	Rlv	0	76,92	0
	Rlt	0	0	100
*nod*D	*Vaf*	100	7,69	0
	Rlv	0	92,31	0
	Rlt	0	0	100
*nif*H	*Vaf*	95,45	23,08	0
	Rlv	4,55	76,92	0
	Rlt	0	0	100

Table S3. Coefficient of nucleotide differentiation between groups *Vaf*, *Rlv* and *Rlt*, Nucleotide distances with standard error within and between groups *Vaf*, *Rlv*, and *Rlt* were calculated in MEGA X.

	coefficient of differentiation						nucleotide diversity within groups						nucleotide diversity between groups					
	Vaf-Rlv		*Vaf-Rlt*		*Rlv-Rlt*		*Vaf*		*Rlv*		*Rlt*		*Vaf-Rlv*		*Vaf-Rlt*		*Rlv-Rlt*	
	d	S.E.	d	S.E.	d	S.E.	d	S.E.	d	S.E.	d	S.E.	d	S.E.	d	S.E.	d	S.E.
16S rRNA	0,044	0,038	0,154	0,020	-0,002	0,027	0,002	0,001	0,002	0,001	0,001	0,000	0,002	0,001	0,001	0,001	0,001	0,001
*dna*K	0,024	0,024	-0,039	0,015	0,051	0,011	0,023	0,005	0,025	0,005	0,051	0,007	0,026	0,006	0,042	0,008	0,043	0,008
*glt*A	0,073	0,023	0,002	0,011	0,045	0,010	0,028	0,004	0,023	0,004	0,039	0,005	0,029	0,004	0,037	0,005	0,034	0,004
*gln*II	0,047	0,015	-0,024	0,008	0,074	0,012	0,033	0,004	0,033	0,004	0,052	0,005	0,038	0,005	0,047	0,005	0,050	0,006
hkg (concatenate)	0,053	0,012	-0,011	0,007	0,057	0,007	0,016	0,001	0,015	0,001	0,025	0,002	0,017	0,001	0,022	0,001	0,022	0,001
*nod*A	0,065	0,027	0,574	0,024	0,570	0,022	0,032	0,004	0,046	0,005	0,095	0,007	0,050	0,007	0,306	0,034	0,307	0,034
*nod*C	0,151	0,029	0,586	0,018	0,567	0,016	0,012	0,002	0,039	0,004	0,102	0,005	0,044	0,006	0,290	0,044	0,301	0,045
*nod*D	0,318	0,031	0,500	0,018	0,495	0,015	0,017	0,003	0,046	0,004	0,139	0,007	0,075	0,024	0,360	0,203	0,367	0,210
*nif*H	0,208	0,057	0,612	0,022	0,573	0,021	0,007	0,002	0,027	0,004	0,080	0,006	0,033	0,005	0,230	0,022	0,222	0,020
sym (concatenate)	0,208	0,020	0,560	0,010	0,544	0,009	0,016	0,001	0,040	0,002	0,106	0,003	0,049	0,003	0,235	0,006	0,237	0,006

Table S4. Average divergence within host specificity groups inferred from Jackknife (JK) data. *CAD = 100% – JK (from Table S2).

Analysed groups	Coefficients of Average Divergence (CAD)*		t-Student (P_0)
	hkg	*sym*	
Vaf	22,54±7,63	1,16±1,13	2,77 (< 0,05)
Rlv	35,90±4,53	17,31±3,68	3,19 (< 0,05)
Rlt	54,16±6,29	0	8,61 (< 0,001)

Figure S1. Set of neighbour joining trees for core and symbiotic loci of *Vaf* (three shades of green corresponding to geographical origin), *Rlv* (yellow) and *Rlt* (pink) groups. The evolutionary distances were computed using the maximum composite likelihood method. Values of bootstrap test (1000 replicates) exceeding 0.5 are shown next to the branches. * presence of *nod*X gene in a strain.

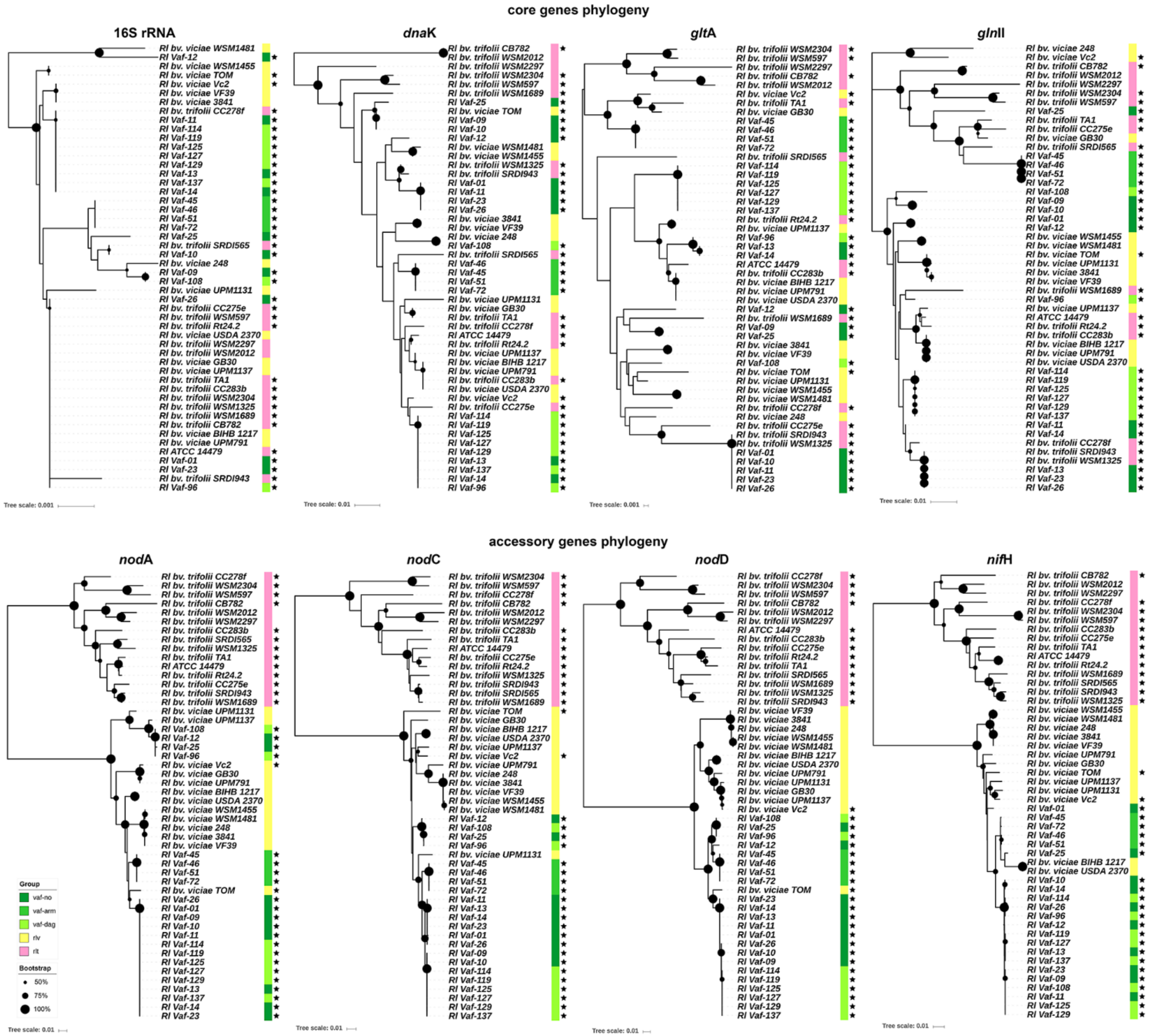

Supplement: Supplementary file 1 [file genes-10-00991-s001.pdf]
